# Effectiveness of mental simulation practices after total knee arthroplasty in patients with knee osteoarthritis: A systematic review and meta-analysis of randomized controlled trials

**DOI:** 10.1371/journal.pone.0269296

**Published:** 2022-06-03

**Authors:** Ting-Hsuan Lee, Chia-Hung Liu, Pei-Chi Chen, Tsan-Hon Liou, Reuben Escorpizo, Hung-Chou Chen

**Affiliations:** 1 Department of Family Medicine, Shuang Ho Hospital, Taipei Medical University, New Taipei City, Taiwan; 2 Department of Family Medicine, School of Medicine, College of Medicine, Taipei Medical University, Taipei, Taiwan; 3 Department of Physical Medicine and Rehabilitation, Shuang Ho Hospital, Taipei Medical University, New Taipei City, Taiwan; 4 Department of Physical Medicine and Rehabilitation, School of Medicine, College of Medicine, Taipei Medical University, Taipei, Taiwan; 5 Department of Rehabilitation and Movement Science, University of Vermont, College of Nursing and Health Sciences, Burlington, VT, United States of America; 6 Swiss Paraplegic Research, Nottwil, Switzerland; 7 Center for Evidence-Based Health Care, Shuang Ho Hospital, Taipei Medical University, New Taipei City, Taiwan; Mugla Sitki Kocman Universitesi, TURKEY

## Abstract

Mental simulation practices, such as motor imagery, action observation, and guided imagery, have been an intervention of interest in neurological and musculoskeletal rehabilitation. Application of such practices to postoperative patients in orthopedics, particularly after total knee arthroplasty, has resulted in favorable physical function outcomes. In this systematic review and meta-analysis, we wish to determine the effectiveness of mental simulation practices with standard physical therapy compared to standard physical therapy alone in patients who underwent total knee arthroplasty in terms of postoperative pain, physical functions, and patient-reported outcome measures. We identified randomized controlled trials from inception to August 28, 2021, by using the PubMed, Cochrane Library, EMBASE, and Scopus databases. Data collection was completed on August 28, 2021. Finally, eight articles (249 patients) published between 2014 and 2020 were included. The meta-analysis revealed that mental simulation practices caused more favorable results in pain [standardized mean difference = −0.42, 95% confidence interval (CI) (−0.80 to −0.04), P = 0.03], range of motion [0.55, 95% CI (0.06–1.04), P = 0.03], maximal strength of quadriceps [1.21, 95% CI (0.31–2.12), P = 0.009], and 36-Item Short-Form Survey [0.53, 95% CI (0.14–0.92), P = 0.007]. Our data suggest that mental simulation practices may be considered adjunctive to standard physiotherapy after total knee arthroplasty in patients with knee osteoarthritis.

## Introduction

Osteoarthritis is a leading cause of disability in adults and frequently affects the knee. When nonsurgical interventions fail to provide desired results in patients with advanced knee osteoarthritis, total knee arthroplasty (TKA) becomes the treatment of choice for ameliorating pain and improving mobility, function, and health-related quality of life [1, 2]. Although TKA offers long-term benefits [2], the immediate and subacute postoperative period is often associated with pain [3], reduced range of motion [4], impairment of quadriceps strength [5], and decreased physical and social functioning [6].

Traditional postoperative rehabilitation protocols have mainly focused on conventional physiotherapy. Several modalities and techniques, such as hydrotherapy [7], cryotherapy [8], neuromuscular electrical stimulation [9], and continuous passive motion therapy [10]. However, while some showed positive effects [9, 11], the clinical benefits of others remain uncertain [4, 12, 13], and there remains an unmet need for innovative post-TKA rehabilitation approaches that can provide significant clinical efficacy.

Over the last two decades, cognitive-based strategies, such as motor imagery and action observation, have attracted attention for motor rehabilitation in patients with stroke [14] and Parkinson’s disease [15, 16], and for the relief of musculoskeletal pain [17, 18]. Motor imagery is a dynamic mental state in which an individual is given a motor movement for mental rehearsal without overt motor output [19]. It is a cognitive simulation process that employs motor task imagery for activating brain regions associated with movement preparation and execution [20–22]. This process is based on the motor simulation theory, which suggests neural networks are involved during imagined and executed movement [22]. Indeed, many studies have demonstrated an overlap in neural activation during actual physical movements and motor imagery [23–25]. Because no movements are performed in the process, motor imagery elicits no pain or other negative side effects that might be linked to routine physical therapy [26, 27]. Another modality of mental simulation practice that is commonly used is action observation, which requires patients to visually perceive specific motor gestures, thereby evoking internal motor simulation [28]. Evidence of the mirror neuron system has suggested that the group of neurons that are activated during certain motor acts are also activated when the same or similar movement is observed [28].

Recently, several randomized controlled trials (RCTs) have explored the effects of these mental stimulation practices on patients after TKA. A meta-analysis concluded that mental simulation practices exert positive effects on physical function in patients after total hip arthroplasty (THA) or TKA [29]; but the included evidence had generally high heterogeneity. Moreover, the effect of post-TKA mental simulation intervention could not be isolated in the previous study because its pooled data included both patients with THA and those with TKA, and only four trials targeting patients with TKA were included. The present systematic review and meta-analysis included RCTs to analyze the effectiveness of mental simulation practices on pain, physical function, and other composite measures in patients after TKA.

## Methods

### Study design and registration

This systematic review and meta-analysis was conducted in accordance with the Preferred Reporting Items for Systematic Reviews and Meta-analysis (PRISMA) guidelines [30]. The protocol was registered in the prospective international register of systematic reviews, PROSPERO (registration number: CRD42021293408)

### Search strategy

A literature search for scientific articles on the effectiveness of mental simulation practices in patients who had undergone TKA was performed using PubMed, Cochrane Library, EMBASE and Scopus databases from inception to August 28, 2021. The following keywords or their combinations were employed for the search: “motor imagery,” “guided imagery,” “mental simulation,” “MSP,” “mental practice,” “action observation,” “AO,” “AOT,” “total knee arthroplasty,” “TKA,” “OA knee,” and “osteoarthrit*”. Search filters of the databases were used to identify RCTs if applicable. No language restrictions were applied. The applied search strategies of the databases can be found in **S1 Table**. The reference lists of the relevant articles were manually reviewed for additional studies. The retrieved RCTs were imported into EndNote X9 (Clarivate Analytics) software and screened for relevance by two reviewers independently; first based on title and abstract, and then by reviewing the full text. Any discrepancies were resolved through discussion with a third reviewer.

### Eligibility criteria

We included RCTs that deployed mental simulation practices, such as motor imagery, action observation or guided imagery, alone or in combination with standard physical therapy (SPT) in patients who underwent TKA for knee osteoarthritis with outcomes of pain, physical function, or patient self-reported measures assessed. We excluded RCTs that used treatment combinations that precluded the isolation of the effectiveness of mental simulation intervention.

### Data extraction

The following parameters were extracted from each RCT by two reviewers independently: the mean age, sex, and number of participants; the type of mental simulation practice used as intervention and description on duration, frequency, and method of delivery; duration and frequency of SPT; details of the placebo/control treatment; appraised outcome measures; and follow-up duration.

### Outcome assessment

The primary outcome measure was pain, assessed using a visual analog scale (VAS). Other outcome measures in relation to physical functions of the lower extremities and composite measures that included activities of daily living, quality of life or general health status were also considered. These included the following: the Timed Up and Go Test (TUG), Barthel Index, the Oxford Knee Score (OKS); and spatiotemporal and kinematic parameters, such as gait speed, cadence, stride length, and range of motion (ROM). Patient-reported outcome measures reflected by questionnaires, specifically the 36-Item Short-Form Health Survey (SF-36) and Western Ontario and McMaster Universities Arthritis Index (WOMAC) scores, were also considered.

### Risk-of-bias assessment

The methodological quality of each study was assessed by two independent reviewers using the Physiotherapy Evidence Database (PEDro) scale [31]. The PEDro scale consists of 11 items that are rated “Yes” or “No” (which corresponds to 1 or 0 points) depending on whether a criterion is met by a study. The ratings of PEDro scale items 2–11 were summed to obtain a total PEDro score between 0 and 10. Scores of <4 were considered “poor,” 4–5 were considered “fair,” 6–8 were considered “good,” and 9–10 were considered “excellent” [32].

### Statistical analysis

Data management and analyses were performed using Review Manager 5.4.1 software (The Cochrane Collaboration, London, United Kingdom). Continuous outcomes measured using different scales were converted to a single scale and expressed as standardized mean differences (SMDs) and 95% confidence intervals (CIs). Data of outcomes that used the same measurement scale were combined as mean difference (MD). A meta-analysis was conducted and presented with a forest plot if two or more trials reported the same outcome. Pooled data were analyzed using a random-effects model for all comparisons. Heterogeneity between studies was investigated using the *I*^2^ statistics, with *I*^2^ > 50% indicating moderate heterogeneity [33]. When the results were statistically significant with a *I*^2^ > 50%, a sensitivity analysis was conducted for the verification of effect. The results were considered statistically significant at *P* ≤ 0.05 in the *z*-tests of equivalence. A funnel plot was used to test for publication bias if 10 or more studies were included in the analysis.

## Results

### Study selection

**Fig 1:**
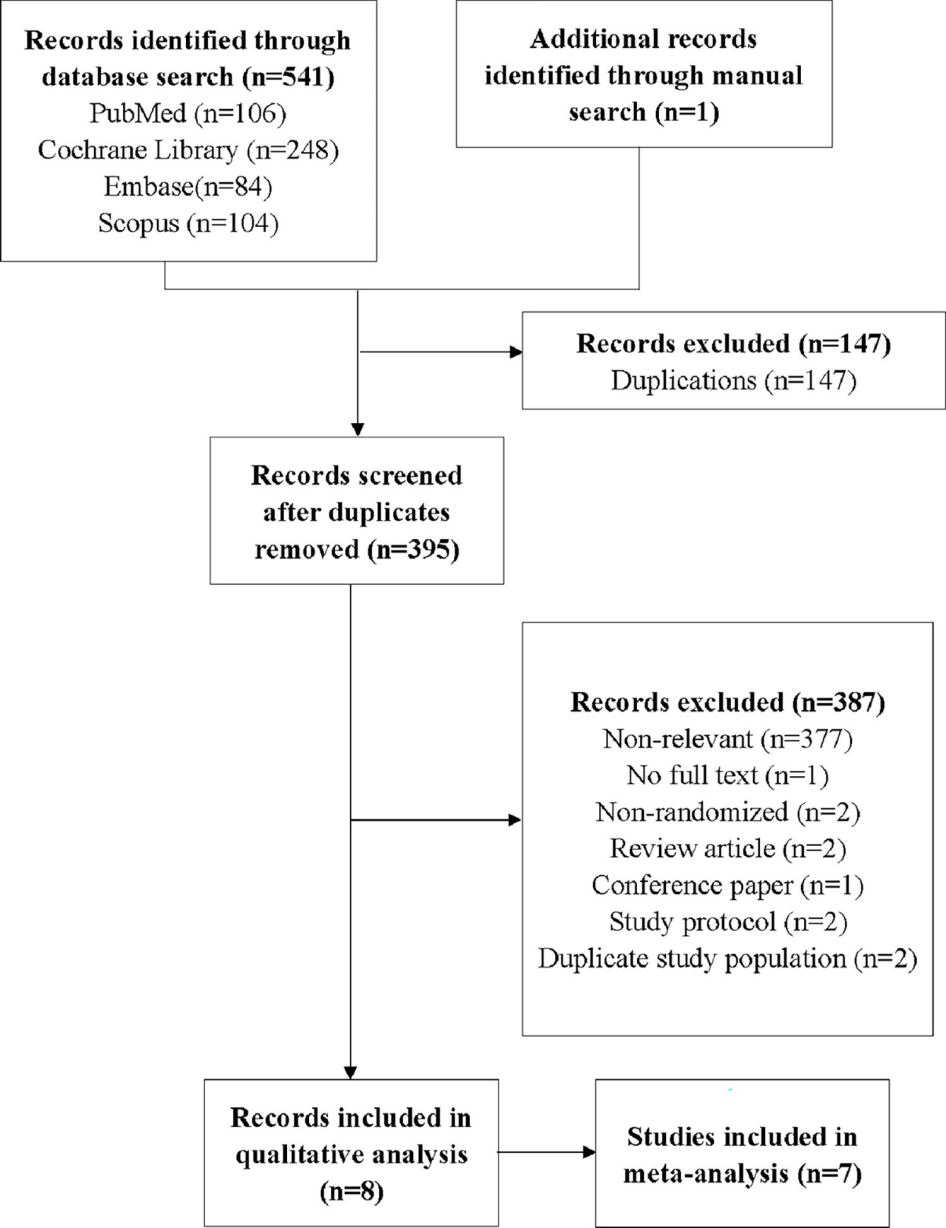


A total of 541 articles were retrieved through the search of electronic databases and one additional article was identified through a manual search of references. Of these studies, 147 were subsequently removed as duplicates and 377 articles were excluded after title and abstract screening. The full texts of the remaining 18 RCTs were screened, of which ten were excluded for various reasons: inaccessibility of full text, nonrandomized study design, review articles, conference paper, study protocols, and not meeting eligibility criteria. Among the eight articles that met the eligibility criteria, two reported different but pertinent outcomes from the same study. Finally, seven RCTs (eight journal articles) [27, 34–40], all parallel studies, comparing the effects of mental simulation practices and SPT on pain and physical function were included in the meta-analysis. A flow chart of the study selection strategy is presented in **Fig 1**.

### Study characteristics

The selected studies were published between 2014 and 2020 and included a total of 249 patients (124 and 125 patients in the intervention and control groups, respectively). Four studies used motor imagery as the choice of mental simulation practice [27, 34–37], two adopted action observation [38, 40] and one used guided imagery [39]. All patients in the intervention and control groups received SPT as the baseline treatment. All studies reported pain as an outcome by using a VAS [27, 34, 35, 37–40]. Five trials assessed ROM by using active knee flexion [27, 34, 35, 37, 38]. Four trials reported TUG [27, 34, 37, 40] by measuring the time taken to complete the walk test. Patient-reported health status, such as WOMAC [35, 39, 40], SF-36 [38, 39], and OKS [27, 37] were each evaluated by two studies. The Barthel Index, a measure of the performance of activities of daily living, was reported by two studies [34, 38]. Spatiotemporal and kinematic parameters, including quadriceps strength [27, 37], gait speed [34, 36], stride length [34, 36], and cadence [34, 36], were each assessed by two studies. The main characteristics of the seven RCTs are summarized in **Table 1**.

**Table 1:** Characteristics of included studies.

Study	Participants				Intervention	Control	Assessed outcome measures
	Experimental group		Control group				
	*n* (men/women)	Mean age (SD)	*n* (men/women)	Mean age (SD)			
Zapparoli et al., 2020 [34]	24 (13/11)	66.2 (8.0)	24 (7/17)	66.6 (7.5)	SPT + MI (2*30 min/d, for an average of 11 d) by visual and audio stimuli	SPT (70 min/d, 6 d/wk) + nonmotorized cognitive training (2*30 min/d, for an average of 11 d)	Pain (VAS), TUG (s), ROM (°), Barthel Index, self-selected gait speed (m/s), gait cadence (steps/min), stride length (m)
Briones-Cantero et al., 2020 [35]	12 (8/4)	72 (6)	12 (7/5)	72 (5)	SPT + MI (30 min/session, 5 sessions in total)	SPT (30 min/session, 5 sessions in total)	Pain (VAS), ROM (°), WOMAC,
Paravlic et al., 2020 [27]	13 (7/6)	61.7 (5.2)	13 (7/6)	58.9.0 (5.2)	SPT + MI (hospitalization: 15 min/d, home: 5 times/wk for 4 weeks via audiotape provided at the time of discharge)	SPT (15 min/d by verbal communication in hospital and by telephone after discharge)	Pain (VAS), TUG (s), ROM (°), MViC (Nm/kg),
Paravlic et al., 2019 [36]	(same as above)	(same as above)	(same as above)	(same as above)	(same as above)	(same as above)	Self-selected gait speed (m/s), gait cadence (steps/min), stride length (m)
Moukarzel et al., 2017 [37]	10 (2/8)	70.3 (2.5)	10 (2/8)	68.9 (1.8)	SPT + MI (15 min/d, 3 d/wk for 4 weeks) by self-visualization of movements	SPT (60 min/d, 3 d/wk for 4 weeks)	Pain (VAS), ROM (°), TUG (s), MViC (Nm/kg),
Jacobson et al., 2016 [39]	42 (31/8)	65.0 (8.6)	40 (19/21)	63.7 (8.8)	SPT + GI (19−21 min/d, 7 d/wk for 5 weeks) by audio-recordings	SPT (17−21 min/d, 7 d/wk for 5 weeks) by audio-recordings	Pain (VAS), WOMAC, WOMAC-Stiffness, WOMAC-Function, SF 36-physical function, SF 36-mental health
Villafañe et al., 2016 [38]	14 (7/7)	70.4 (7.5)	17 (3/14)	70.1 (7.7)	SPT + AO (2 sessions/d, 5 d/wk for 2 weeks) by a video of exercises being performed	SPT (2*30 min/d, 5 d/wk for 2 weeks) + video of scenes in nature	Pain (VAS), ROM (°), Barthel Index, SF 36-physical function, SF 36-mental health
Park et al., 2014 [40]	9 (NA)	72.7 (12.3)	9 (NA)	70.6 (11.0)	SPT + AO (10 min/d, 3 d/wk for 3 weeks) by video clip	SPT (30 min/d, 3 d/wk for 3 weeks)	Pain (VAS), TUG (s), WOMAC-Stiffness, WOMAC-Function,

AO, Action observation; d, day(s); GI, Guided imagery; MI, Motor imagery; min, minute(s); MViC, Maximal voluntary isometric contraction; *n*, number; NA, Not applicable; ROM, Range of motion; s, second(s); SF-36, 36-Item Short Form Survey; SD, Standard deviation; SPT, Standard physical therapy; TUG, Timed Up and Go Test; VAS, Visual analogue scale; wk, week; WOMAC, Western Ontario and McMaster Universities Osteoarthritis Index.

### Risk-of-bias assessment results

All studies reported adequate baseline comparability, between-group statistical comparison, point estimates, and variability measures for at least one key outcome. All studies corresponded to random allocation, one study [35] mentioned achievement of concealed allocation. Participants in all the studies were not blinded. Three studies clearly stated blinding of therapists [27, 37, 39] and/or assessors [35, 38, 39]. All studies reported more than 85% follow-up for at least one key outcome. Four studies [27, 35, 37, 38] were analyzed by the intention-to-treat approach.

All PEDro scale scores in the included studies were between 5 and 8; two studies [34, 40] were considered “fair,” five studies [27, 35, 37–39] were considered “good”. The scores of each item on the PEDro scale are summarized in **Table 2**.

**Table 2:** Summary of methodological quality based on PEDro scale.

	PEDro scale items											PEDro score	
Studies included	1*	2	3	4	5	6	7	8	9	10	11	(0–10)	Methodological quality
Briones-Cantero, 2020	Y	Y	Y	Y	N	N	Y	Y	Y	Y	Y	8	Good
Jacobson, 2016	Y	Y	N	Y	N	Y	Y	Y	N	Y	Y	7	Good
Moukarzel, 2017	N	Y	N	Y	N	Y	N	Y	Y	Y	Y	7	Good
Paravlic, 2020	Y	Y	N	Y	N	Y	N	Y	Y	Y	Y	7	Good
Park, 2014	Y	Y	N	Y	N	N	N	Y	N	Y	Y	5	Fair
Villafañe, 2016	N	Y	N	Y	N	N	Y	Y	Y	Y	Y	7	Good
Zapparoli, 2020	Y	Y	N	Y	N	N	N	Y	N	Y	Y	5	Fair

**Items**: 1- Eligibility criteria specified; 2-Random allocation; 3-Concealed allocation; 4-Baseline comparability; 5-Blinded participants; 6-Blinded therapists; 7-Blinded assessors; 8-Adequate follow-up; 9-Intention-to-treat analysis; 10-Between-group comparisons; 11-Point estimates and variability. *Not included in the calculation of the total score

**Methodological quality**: Excellent, 9–10 points; Good, 6–8 points; Fair, 4–5points; Poor, 0–3 points; Yes (**Y**), 1 point; No (**N**), 0 point.

### Knee pain

**Fig 2:**
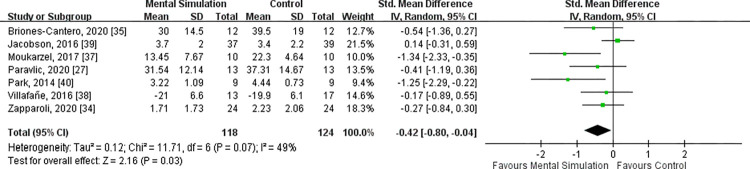


All studies [27, 34, 35, 37–40] reported VAS scores of the targeted knee at the acute to subacute period after surgery, ranging from 10 days to 4 weeks post-TKA. Heterogeneity existed between the seven trials (*I*^2^ = 49%, P = 0.07); hence, a random-effects model was adopted. The meta-analysis revealed that the pain score was significantly lower in the mental simulation practice group than in the control group [SMD = −0.42, 95% CI (−0.80 to −0.04), P = 0.03] (**Fig 2**).

### ROM

**Fig 3:**
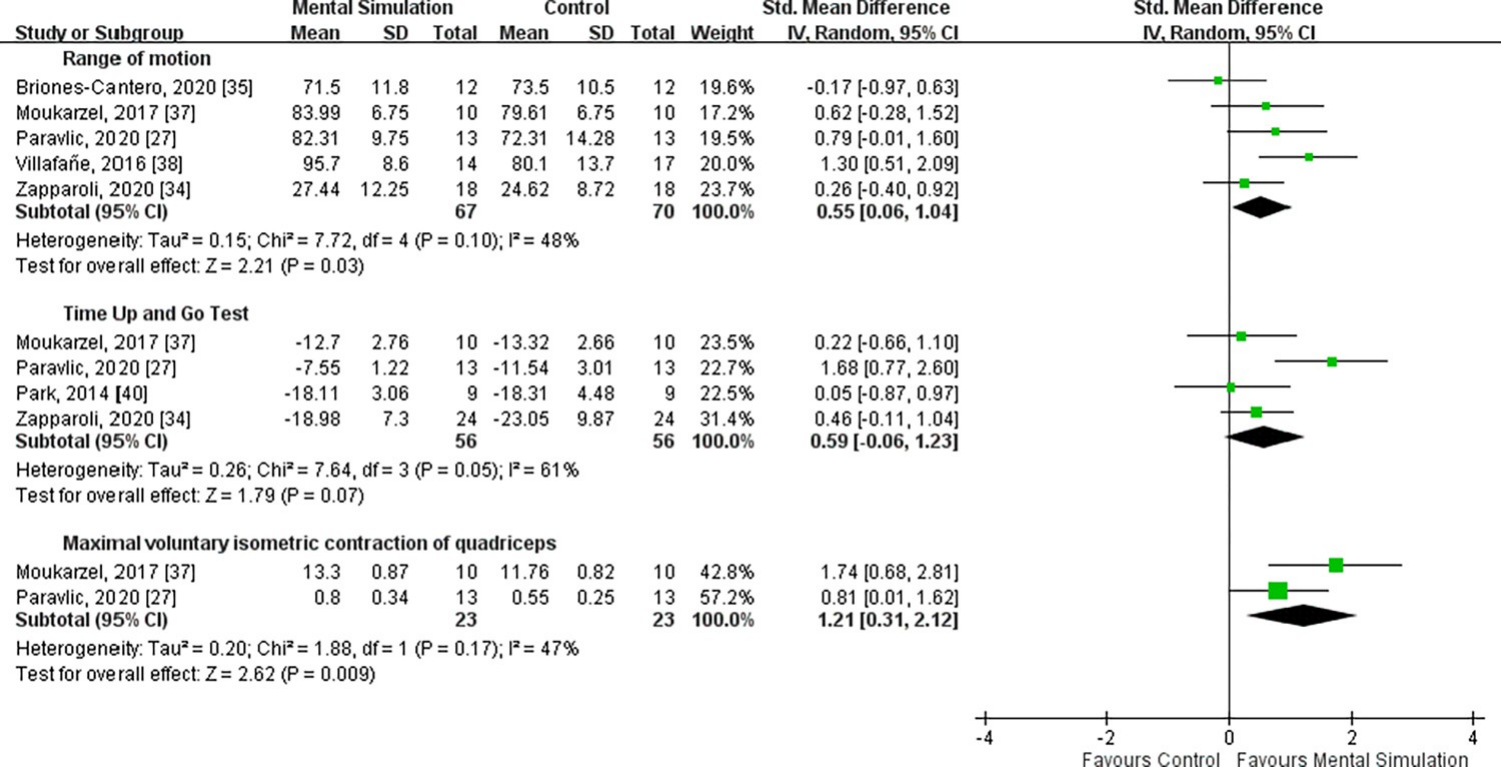


ROM was reported in five studies [27, 34, 35, 37, 38], with a total of 137 patients (67 in the mental simulation practice group and 70 in the control group). Heterogeneity was considered moderate (*I*^2^ = 48%, P = 0.10). The overall pooled data suggested that mental simulation practice can significantly improve active knee flexion range of motion in patients after TKA [SMD = 0.55, 95% CI (0.06−1.04), P = 0.03] (**Fig 3**).

### Muscle strength

The voluntary isometric contraction of knee extensors on the operated leg was assessed by two studies [27, 37], with 23 patients each in the mental simulation practice and control groups. Moderate heterogeneity was observed between the two studies (*I*^2^ = 47%, P = 0.17). The results indicated that compared with the control group, mental simulation practice was associated with significantly improved quadriceps strength [SMD = 1.21, 95% CI (0.31−2.12), P = 0.009] (**Fig 3**).

### TUG test

Four of the RCTs [27, 34, 37, 40], including 112 patients (56 each in the mental simulation practice and control groups), provided data on the Timed Up and Go Test (TUG). Moderate heterogeneity was observed among the studies (*I*^2^ = 61%, P = 0.07). No significant difference was observed in the TUG between the mental simulation practice group and control group [SMD = 0.59, 95% CI (−0.06 to 1.23), P = 0.07] (**Fig 3**).

### WOMAC scores

Three studies compared the WOMAC score; of them, two studies assessed the total WOMAC scores (49 and 51 patients in the mental simulation practice group and control groups, respectively) [35, 39], and two assessed the function and stiffness subscales (46 and 48 participants in the experimental and control groups, respectively) [39, 40].

**Fig 4:**
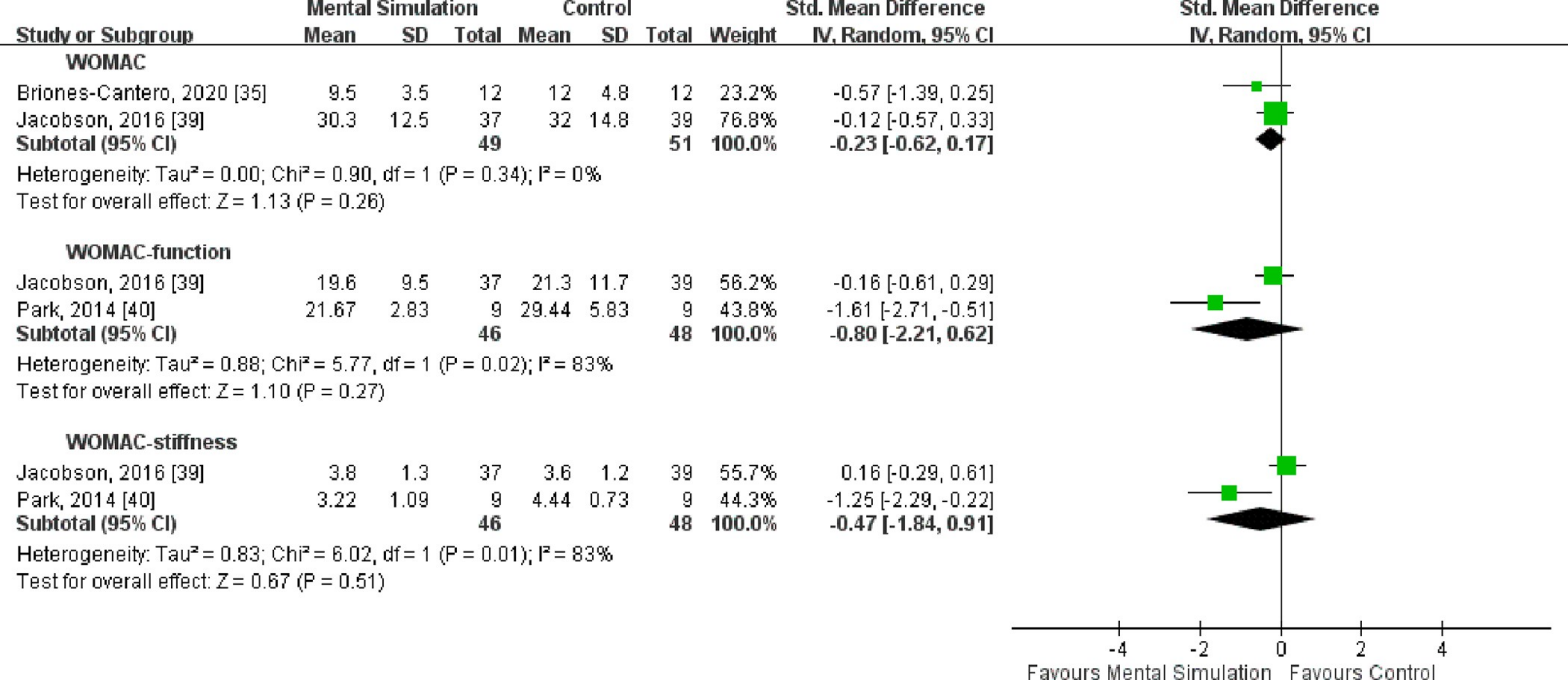


No heterogeneity was observed in the two studies that compared total WOMAC scores (*I*^2^ = 0%, P = 0.34). The overall estimate revealed no significant difference between the mental simulation practice and control groups in total WOMAC score [SMD = −0.23, 95% CI (−0.62 to 0.17), P = 0.26] (**Fig 4**).

Pooled results revealed no significant difference between the experimental and control groups in function and stiffness subscale scores [SMD = −0.80, 95% CI (−2.21 to 0.62), P = 0.27 for functional score, SMD = −0.47, 95% CI (−1.84 to 0.91), P = 0.51 for stiffness score]. Significant heterogeneity was noted in both comparisons (functional: *I*^2^ = 83%, P = 0.02 and stiffness: *I*^2^ = 83%, P = 0.01) (**Fig 4**).

### 36-Item Short Form Health Survey

**Fig 5:**
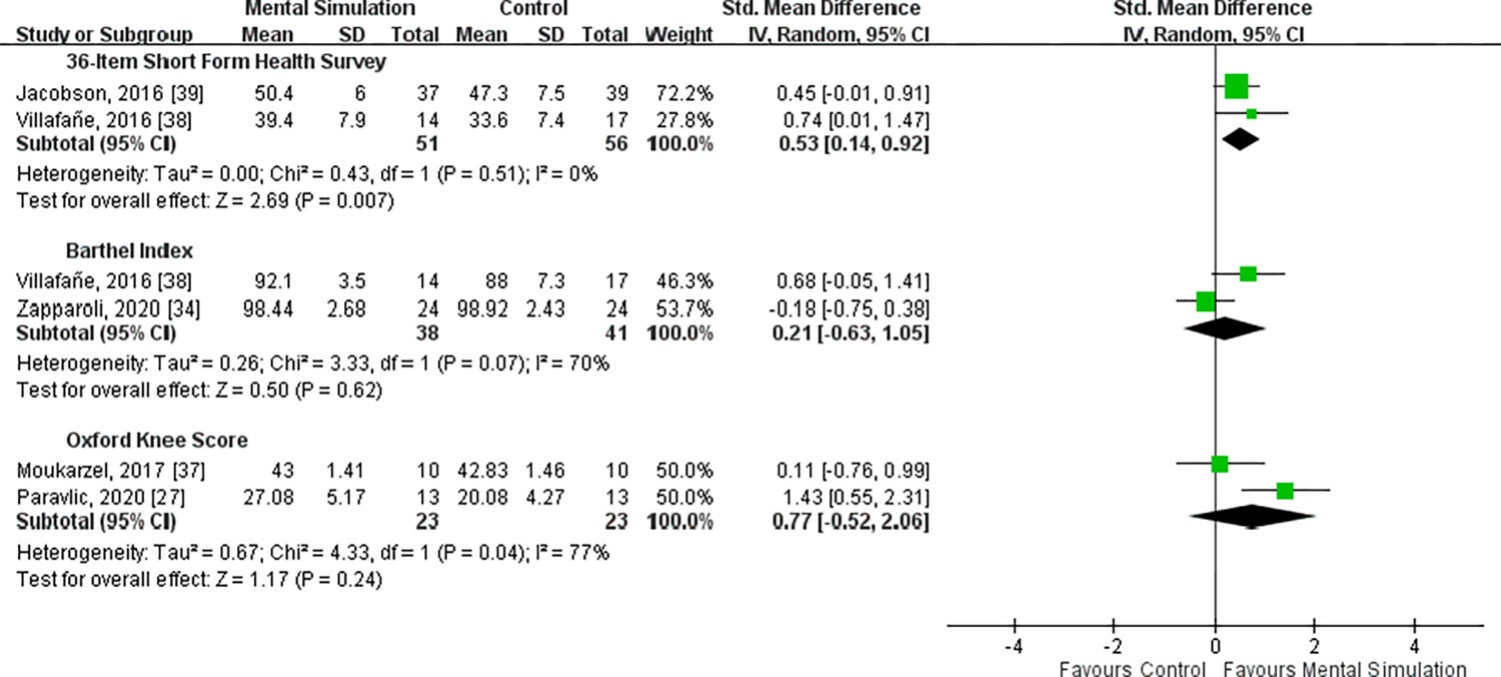


Two studies assessed patient-reported health status by using the SF-36 questionnaire [38, 39]. No heterogeneity was found between the two trials (*I*^2^ = 0%, P = 0.51). The results demonstrated a significant improvement in SF-36 scores in the mental simulation group compared with the control group [SMD = 0.53, 95% CI (0.14 to 0.92), P = 0.007] (**Fig 5**).

### Barthel Index

The two studies that explored Barthel Index scores showed high heterogeneity (*I*^2^ = 70%, P = 0.07) [34, 38], and the pooled data exhibited no significant difference between the intervention and control groups [SMD = 0.21, 95% CI (−0.63 to 1.05), P = 0.62] (**Fig 5**).

### OKS

Two studies used the OKS [27, 37]. No significant difference was noted between the two groups [SMD = 0.77, 95% CI (−0.52 to 2.06), P = 0.24]. Substantial heterogeneity was also observed between the two trials (*I*^2^ = 77%, P = 0.04) (**Fig 5**).

### Gait parameters

**Fig 6:**
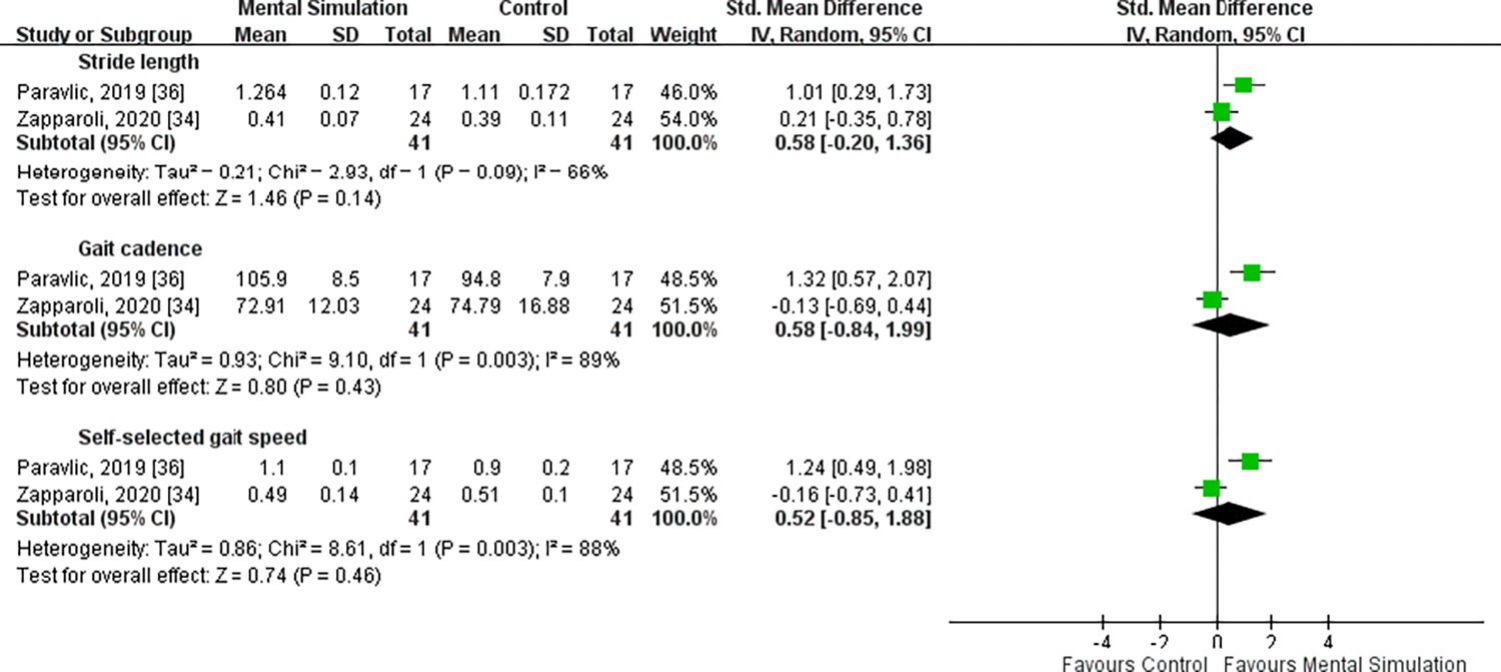


Stride length, cadence, and gait speed were evaluated by two studies with moderate to substantial heterogeneity (*I*^2^ = 66%, P = 0.09 for stride length; *I*^2^ = 89%, P = 0.003 for cadence; and *I*^2^ = 88%, P = 0.003 for gait speed) [34, 36]. In all three comparisons, no significant difference was found between the mental simulation practice and control group [SMD = 0.58, 95% CI (−0.20 to 1.36), P = 0.14 for stride length; SMD = 0.58, 95% CI (−0.84 to 1.99), P = 0.43 for cadence; and SMD = 0.52, 95% CI (−0.85 to 1.88), P = 0.46 for gait speed] (**Fig 6**).

### Adverse effect

Given that mental simulation is a cognitive process that involves the mental rehearsal of perceptual information in the absence of actual motor output, it is usually conducted noninvasively, with limited adverse effects and complications [41]. None of the included studies reported any adverse events related to mental simulation intervention.

## Discussion

In this systematic review and meta-analysis, we synthesized evidence of the effectiveness of mental simulation practices, specifically motor imagery, action observation and guided imagery, in pain, patient-reported outcome measures, as well as clinical and kinematic parameters of patients who underwent TKA exclusively. The results indicated that the use of mental simulation practice significantly improved pain, ROM, muscle strength of knee extensors, and SF-36 score but not cadence, gait speed, stride length, Barthel Index, or TUG, WOMAC, and its subscale scores.

The motor simulation theory, which postulates that various action-related cognitive states, such as imagery and observation, activate motor systems in the brain that are similar to those triggered during actual action [21, 42, 43], may provide an explanation for these results. Neuroimaging studies have revealed that both imagined and real movements share a common neural substrate [44], and mental simulation practice may be useful in preventing the loss of physical function in both upper [45] and lower extremities [46]. The proposed underlying mechanisms range from selective modulation of corticospinal system excitability [47] to activation of the mirror neuron system [48]. Although the precise mechanism remains unclear [42], discussion on mental simulation interventions is warranted due to the expected clinical benefits.

Discussion on the representation and simulation of motor acts dates back to as early as 1825 by the German philosopher and psychologist, Johann Friedrich Herbart, who proposed that the imagery of perceptual effects can elicit related movements [49]. It has since then been explored in various aspects of sports [50] and medicine [14–18, 51]. Mental simulation practice has been used to rehabilitate motor deficits in various neurological [52] and musculoskeletal disorders [17, 18, 51]. A systematic review and meta-analysis reported multiple positive effects on measures of physical function recovery compared with SPT in patients after TKA or THA [29]. Positive effects were observed in the maximal strength of knee extensors and various mobility measures, including gait speed, TUG, joint flexion, and joint extension assessments. For overall physical function, the study collectively assessed numerous variables by using composite effect size which has also shown favorable effects. However, substantial heterogeneity within effect estimates of several outcomes was noted. The study also did not include an assessment of important patient-subjective outcomes, such as pain.

Increasing evidence suggests that mental simulation can decrease pain in various situations of musculoskeletal pain [17, 18], including postamputation phantom limb pain [41] and complex regional pain syndrome [53]. Despite successful joint replacement, many people continue to experience significant pain and functional problems. A systematic review concluded that unfavorable long-term pain outcomes were seen in 10%−34% of patients after knee arthroplasty [54]. Our data revealed that mental simulation practice significantly reduces early to intermediate-term postoperative pain (SMD, −0.42), as evidenced by VAS, in patients who have endured TKA. Considering that several studies have reported positive associations between intermediate-term postoperative pain and long-term pain outcomes [55, 56], mental simulation practices may be useful in reducing intermediate-phase postoperative pain, thereby potentially leading to improved longer-term pain control.

TKA alleviates pain and improves mobility, but functional deficits may persist in the long term [6] compared with healthy adults; for example, a study demonstrated 18% and 51% slower walking and stair-climbing speed, respectively, and approximately 40% deficits in quadriceps strength in patients after TKA [57]. Our data indicated significant differences in the active ROM and quadriceps strength of the operated leg with moderate heterogeneity after application of mental simulation practice to SPT. No significant improvements in TUG, gait speed, cadence, and stride length were observed after the implementation of mental simulation techniques. The conflicting finding in TUG of the previous and current meta-analysis may be because the previous meta-analysis assessed combined results of TKA and THA, as TUG requires both hip and knee functionality. Nevertheless, pooled data indicated that mental simulation training may improve gait and motor performance, as represented by a faster TUG.

This study also included various tools to comprehensively appraise different perspectives of mental simulation effects. Aside from SF-36, no significant improvement was noted in any other composite measures—the Barthel Index, WOMAC, WOMAC-function, WOMAC-stiffness, and OKS—after mental simulation intervention was added to SPT. This may be due to the limited number of studies reporting these outcomes (two each). Nevertheless, assessment of these composite outcomes revealed that mental simulation practices may improve quality-of-life measures, represented by SF-36, with limited effect on activities of daily living, functional mobility, gait and general health explored by the Barthel Index, WOMAC, WOMAC-function, WOMAC-stiffness, and OKS.

This study has several limitations. First, owing to the nature of the intervention, therapist and assessor blinding were not conducted in some studies. Patient blinding was unachievable in all included trials, potentially leading to performance bias. Second, a relatively small sample size was investigated across the studies, which may have limited the strength of the results. Third, mental simulation protocols varied amongst the included trials, which may have resulted in the moderate-to-substantial heterogeneity observed across some trials. Fourth, the follow-up duration in the included studies was not sufficiently long for assessing long-term outcomes, and the different follow-up durations may have contributed to bias. Finally, our findings might not apply to patients who underwent TKA for reasons other than knee osteoarthritis. Further larger-scale studies with high methodological quality are required to determine the optimal mental simulation practice protocol and its long-term effects.

## Conclusion

In this systematic review and meta-analysis of RCTs of patients with knee osteoarthritis who underwent TKA, mental simulation practices were noted to produce beneficial effects on pain, ROM, maximal quadriceps strength, and general health status expressed by the scores of SF-36 in the intermediate postoperative period compared with SPT alone, with no adverse effects. Although mental simulation interventions may be considered adjunctive to standard postoperative physiotherapy in patients with knee osteoarthritis after TKA, future research is needed for the investigation of its long-term effects.

## Supporting information

S1 Table Search strategy table. (DOCX)

S1 Fig PRISMA checklist. (DOCX)
