# Supplementary material for: Effectiveness of mental simulation practices after total knee arthroplasty in patients with knee osteoarthritis: A systematic review and meta-analysis of randomized controlled trials
Source: PLoS One. 2022 Jun 3;17(6):e0269296. doi: 10.1371/journal.pone.0269296 (PMC9165806; doi:10.1371/journal.pone.0269296)
Supplement: S1 Table — (DOCX) [file pone.0269296.s001.docx]

| **S1 Table.** Search Strategy Table | | |
| --- | --- | --- |
| **Database** | **Search strategy** | **Results** |
| PubMed | (motor imagery OR guided imagery OR mental simulation OR MSP OR mental practice OR action observation OR AO OR AOT) AND (total knee arthroplasty OR TKA OR OA knee OR osteoarthrit*)  Filter: randomized controlled trial | 106 |
| Cochrane Library | (motor imagery OR guided imagery OR mental simulation OR MSP OR mental practice OR action observation OR AO OR AOT) AND (total knee arthroplasty OR TKA OR OA knee OR osteoarthrit*)  Filter: trials | 248 |
| Embase | ('motor imagery'/exp OR 'motor imagery' OR (('motor'/exp OR motor) AND ('imagery'/exp OR imagery)) OR 'guided imagery'/exp OR 'guided imagery' OR (guided AND ('imagery'/exp OR imagery)) OR 'mental simulation' OR (mental AND ('simulation'/exp OR simulation)) OR msp OR 'mental practice'/exp OR 'mental practice' OR (mental AND ('practice'/exp OR practice)) OR 'action observation'/exp OR 'action observation' OR (('action'/exp OR action) AND ('observation'/exp OR observation)) OR ao OR aot) AND ('total knee arthroplasty'/exp OR 'total knee arthroplasty' OR (('total'/exp OR total) AND ('knee'/exp OR knee) AND ('arthroplasty'/exp OR arthroplasty)) OR tka OR 'oa knee' OR (oa AND ('knee'/exp OR knee)) OR osteoarthrit*)  Filter: randomized controlled trial | 84 |
| Scopus | ALL((motor AND imagery OR guided AND imagery OR mental AND simulation OR msp OR mental AND practice OR action AND observation OR ao OR aot) AND (total AND knee AND arthroplasty OR tka OR OA AND knee OR osteoarthrit*) ) | 104 |
